# The role of extracellular vesicles in COPD and potential clinical value

**DOI:** 10.1186/s12931-024-02719-z

**Published:** 2024-02-08

**Authors:** Shasha Liu, Xiaowu Tan, Sha Liu

**Affiliations:** Department of Pulmonary and Critical Care Medicine, The Second Affiliated Hospital, Hengyang Medical School, University of South China, Hengyang, Hunan China

**Keywords:** Extracellular vesicles, Chronic obstructive pulmonary disease, Pathogenesis, Biomarkers, Clinical application

## Abstract

Chronic obstructive pulmonary disease (COPD) is a heterogeneous lung disease and a major health burden worldwide. Extracellular vesicles (EVs) are nanosized vesicles which possess a lipid bilayer structure that are secreted by various cells. They contain a variety of bioactive substances, which can regulate various physiological and pathological processes and are closely related to the development of diseases. Recently, EVs have emerged as a novel tool for intercellular crosstalk, which plays an essential role in COPD development. This paper reviews the role of EVs in the development of COPD and their potential clinical value, in order to provide a reference for further research on COPD.

## Introduction

Chronic Obstructive Pulmonary Disease (COPD) is a heterogeneous lung condition characterized by chronic respiratory symptoms (dyspnea,cough, sputum production) due to abnormalities of the airways (bronchitis, bronchiolitis) and/or alveoli (emphysema) that cause persistent, often progressive, airflow obstruction [1].COPD is a major health burden across the world. According to statistics provided by the World Health Organization (WHO), COPD has emerged as the third leading cause of mortality globally, accounting for approximately 3.23 million deaths in 2019 [2].A large cross-sectional study conducted in China in 2018 by China Pulmonary Health (CPH) revealed that the prevalence rate of COPD among individuals aged 40 years and above was 13.7%, which translates to a total of almost 100 million people [3]. This is a significant increase from the national COPD prevalence rate of 8.2% for those aged 40 years and older, as reported in 2007 [3].The prevalence of COPD may be further affected by continued exposure to environmental pollution, while the number of smokers worldwide remains high. Total COPD deaths are projected to rise to 8.3 million in 2030 under baseline scenario [4]. And because of the serious disease burden caused by repeated acute exacerbations of the disease, end-stage patients often lose their workforce, which makes the situation of prevention and treatment of COPD still very serious. Therefore, it is imperative to enhance the comprehension of COPD pathogenesis and pinpoint novel therapeutic targets. Extracellular vesicles (EVs) have emerged as a novel tool for intercellular communication and involved in maintaining normal lung homeostasis or responding to pathological developments [5]. EVs have the potential to serve as future novel biomarkers and therapeutics in various diseases, making them valuable for clinical application [6]. The aim of this review is to provide a concise summary of the most recent research findings on the pathological roles of EVs in the development of COPD, as well as to explore their potential applications as both biomarkers and therapeutic interventions.

## Extracellular vesicles

**Fig. 1:**
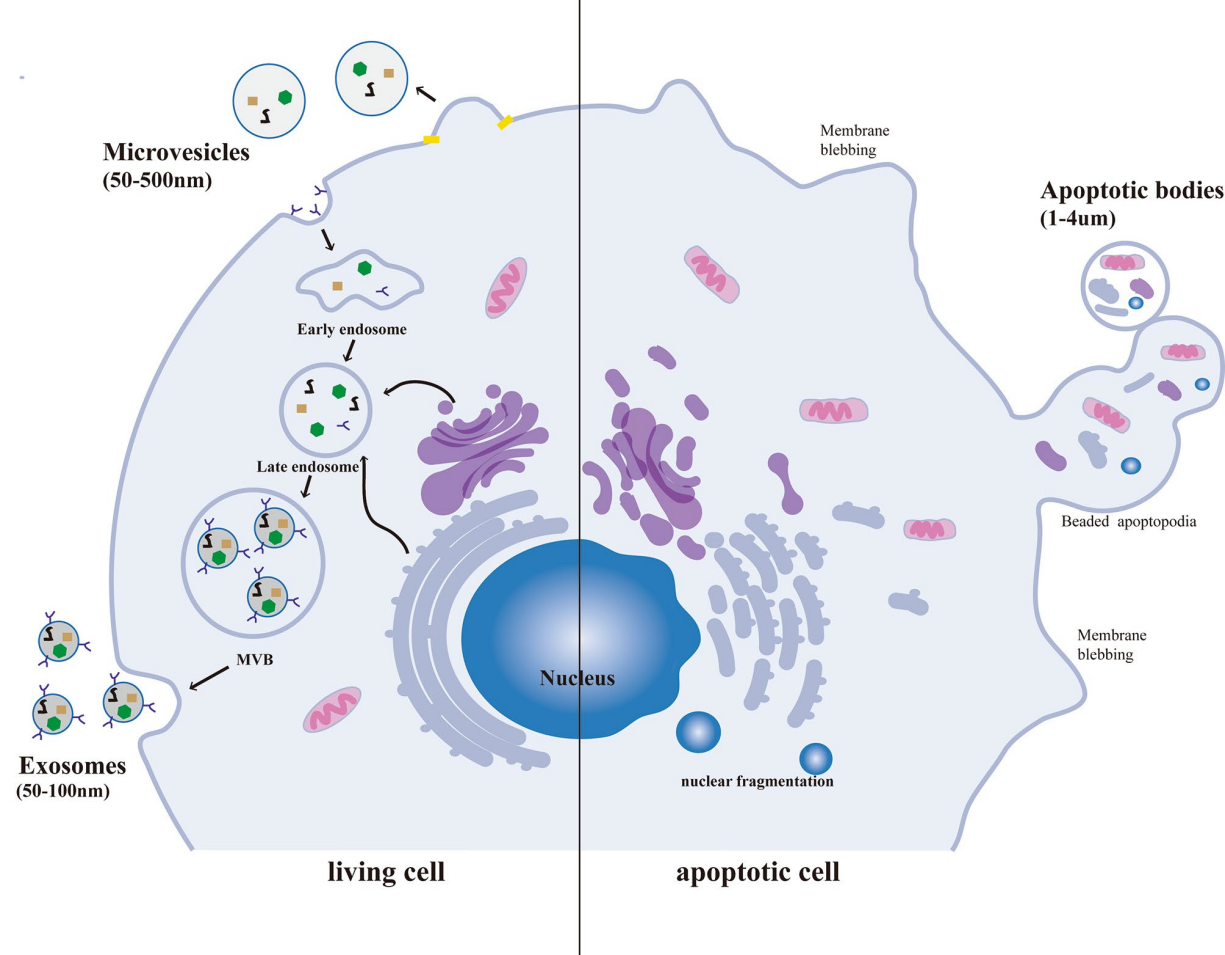
Depiction of EVs subtybes and biogenesis. Exosomes are a type of extracellular vesicle that range in size from 50 to 150 nm and are formed in multivesicular bodies (MVBs) before release in living cell. Microvesicles are a type of extracellular vesicle that range in size from 50 to 500 nm (up to 1 μm) and are larger than exosomes. Microvesicles are released from the cell membrane surface through a process of budding in living cell. Apoptotic bodies, released as blebs of cells undergoing apoptosis, typically fall within a larger size range of 1–4 μm

EVs are membrane-bound vesicles with a lipid bilayer that are secreted by almost all types of cells [7]. EVs play vital roles in the human body, serving as crucial mediators for intercellular communication [8]. Based on size, biogenesis, and secretion mechanism, they are divided into three categories: exosomes, microvesicles, and apoptotic bodies (Fig. 1). Exosomes are a type of extracellular vesicle that range in size from 50 to 150 nm and are released from intracellular vesicles [7]. They are formed in multivesicular bodies (MVBs) before release [9].When the MVB membrane fuses with the cell membrane of the origin, the exosomes are collectively released from the cells into the surrounding environment [9]. Exosomes are rich in specific surface markers, particularly endosomal markers such as CD9, CD63, and CD81 from the tetraspanin protein family, TSG101 (tumor susceptibility gene 101), and Alix [10]. Microvesicles are a type of extracellular vesicle that range in size from 50 to 500 nm (up to 1 μm) and are larger than exosomes [11]. Microvesicles are released from the cell membrane surface through a process of budding [11].Thus, they tend to reflect the surface composition of their parent cells and express cellular markers of the latter [11]. Apoptotic bodies, released as blebs of cells undergoing apoptosis, typically fall within a larger size range of 1–4 μm [12]. Proteins from the plasma membrane, cytosol, as well as fragmented nuclei are present in apoptotic bodies [13]. Apoptotic vesicles are a result of programmed cell death, so the extracellular vesicles involved in regulating intercellular communication mainly refer to exosomes and microvesicles. The International Society of Extracellular Vesicles (ISEV) recommends using the term EVs as a general designation for all nanoparticles released by cells with a lipid bilayer [14]. Unless specifically indicated, the term EVs is used generically for particles in this review.

## EVs in normal airway physiology

EVs have been demonstrated in a diverse range of bodily fluids in the respiratory system, including saliva, sputum and alveolar lavage fluid [15].Under normal pulmonary physiological conditions, EVs play a crucial role in maintaining pulmonary homeostasis by facilitating intercellular communication within the human airway [16]. A diverse array of cells, including lung epithelial cells, endothelial cells, and various immune cells, are capable of releasing EVs. EVs derived from airway epithelial cells contain membrane mucins on their surface, which are part of the mucociliary clearance system and innate immunity [17]. These molecules play a crucial role in protecting the respiratory tract from environmental pathogens [17].EVs derived from alveolar macrophages contain suppressor of cytokine signaling protein 1 (SOCS1) and suppressor of cytokine signaling protein 3 (SOCS3), which regulate inflammatory and maintain alveolar homeostasis [18].In addition, miR-223, an RNA molecule enclosed in EVs secreted by alveolar macrophages, is tansferred to varied cells including lung epithelial cells for the purpose of regulating the airway microenvironment and modulating cellular homeostasis [19]. All of these studies indicate that EVs released from lung cells during normal physiological conditions play a crucial role in maintaining host defense and pulmonary homeostasis. EVs serve as carriers for intercellular signaling and are produced and eliminated in a dynamic equilibrium under physiological conditions [16]. Once external stimuli disturb the homeostasis, EVs can participate in various pathological processes by modulating their target cells [16].

## The role of EVs in the pathogenesis Of COPD

**Fig. 2:**
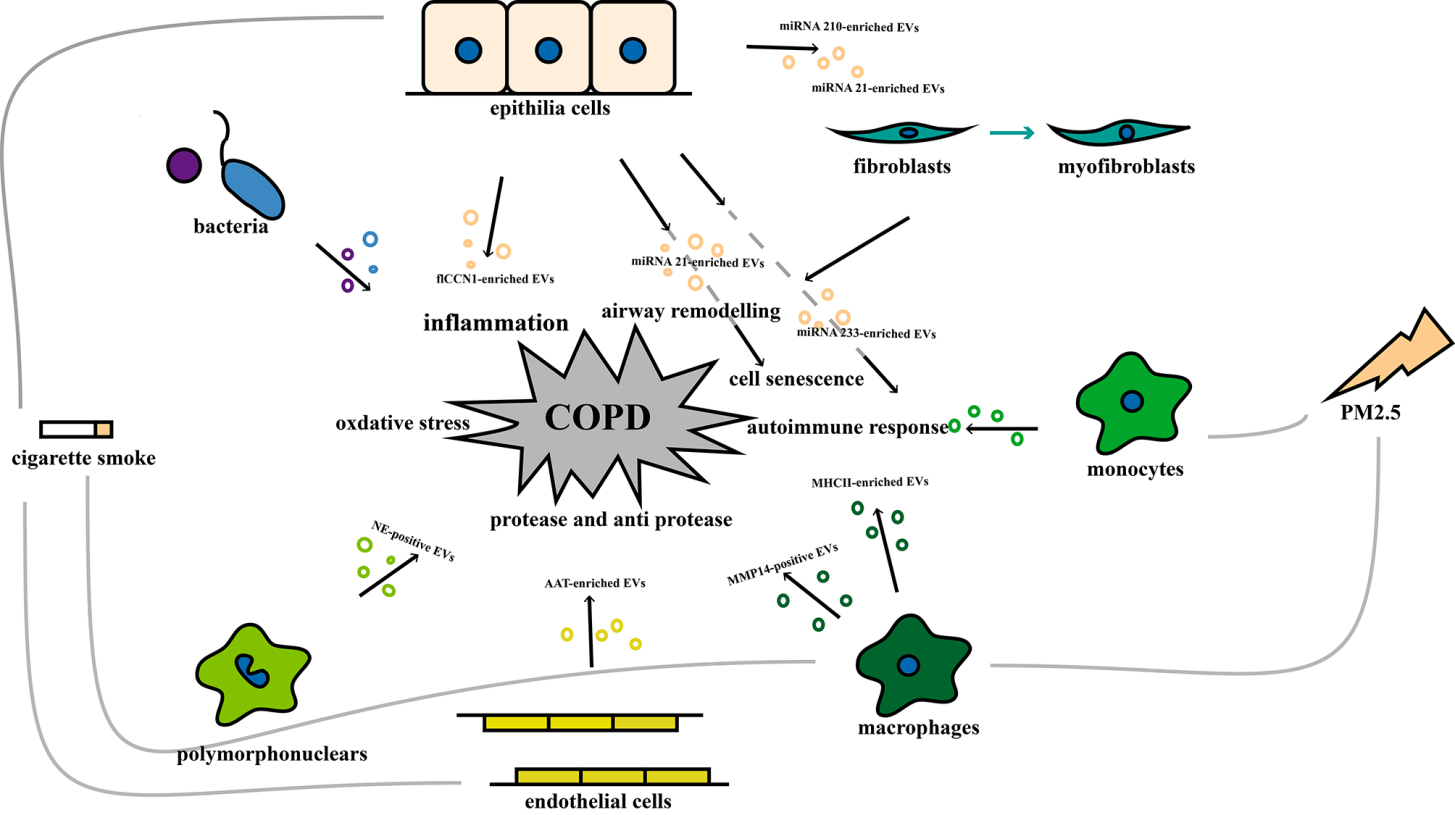
The Role of EVs in The Pathogenesis of COPD. EVs may act as central mediators and contribute to all the mechanisms in COPD pathophysiology including inflammation, protease-anti-protease imbalance, oxidative stress, autoimmune response and cell senescence

The pathogenesis of COPD is complex, involving mechanisms such as inflammation, oxidative stress, and protease and anti-protease imbalance. Recent evidence indicates that both autoimmune responses and microbial changes in the lungs can have an impact on COPD [20].Pathogenic mechanisms do not exist singularly and may predominate in certain processes, but they typically coexist and have a tendency to converge or cycle in order to reinforce one another [21]. EVs may act as central mediators and contribute to all the mechanisms involved in COPD pathophysiology (Fig. 2). We focused on reviewing the role of EVs in the following mechanisms.

### Inflammation

COPD is a complex disease, characterized by inflammation of the airways, destruction of lung tissue, and limited airflow due to changes in the airway structure [22]. EVs act as transporters in the lung, transmitting pro-inflammatory mediators or inflammatory substances to other cells, thereby promoting the pathogenesis of COPD [23–27]. A study revealed that patients with COPD, whether in exacerbation or stable state, had significantly higher levels of plasma exosomes compared to non-smoking healthy individuals [28]. The exosomes were found to be positively correlated with inflammatory markers including C-reactive protein (CRP), soluble tumor necrosis factor type I receptor (sTNFR1), and interleukin-6 (IL-6) [28]. This finding suggests that exosomes may play a role in the inflammatory process of COPD. Moon et al. demonstrated that the secretion of CCN1-enriched exosomes was induced by cigarette smoke extract (CSE) [23].CCN1, also known as CYR61 (cysteine rich 61), belongs to the CCN (CYR61/CTGF/NOV) family of proteins [29].It is an early response gene product that functions as a cysteine-rich extracellular matrix protein involved in various cellular processes such as proliferation, adhesion, migration, differentiation and apoptosis [29]. Additionally, it has been found to be well correlated with inflammatory indicators of disease [29].CCN1 enhanced IL-8 secretion through the Wnt signaling pathway [24]. Furthermore, the increased secretion of IL-8, in turn attracts inflammatory cells, particularly neutrophils, to infiltrate the lung parenchyma [24]. A study conducted by Martin et al. revealed that exposure to PM2.5 resulted in the release of macrophage-derived EVs, which subsequently induced a pro-inflammatory phenotype in lung epithelial cells, leading to the secretion of IL-6 and tumor necrosis factor-alpha (TNFα) [25].Another research conducted by Cordazzo et al. revealed that CSE triggered the release of EVs from monocytes, which in turn stimulated the production of IL-8, monocyte chemotactic protein-1 (MCP-1), and intercellular adhesion molecule-1 (ICAM-1 or CD54) in bronchial epithelial cells [26]. Feller discovered that smoking led to an increase in the expression of Wnt5a and inflammatory cytokines in both mouse models and human specimens [27]. Additionally, EVs transported these factors to other organs in patients with COPD [27].This could potentially explain the systemic inflammatory response observed in individuals with COPD.

One of the causes of exacerbations in COPD patients are EVs derived from bacteria [30],which contribute to disease progression by inducing inflammation. There is evidence to suggest that bacteria have adapted to utilize EVs as contributors to neutrophilic pulmonary inflammation, which plays a role in the pathogenesis of COPD [31, 32]. Kim et al. initially discovered that repeated inhalation of EVs derived from staphylococcus aureus can trigger Th1 and Th17 cell responses, as well as a pulmonary inflammatory response characterized by neutrophil infiltration [31].The inflammatory response induced by S. aureus EVs is primarily dependent on TLR2 signaling [31].In 2015, the same group discovered that EVs derived from Escherichia coli can cause emphysema in an IL-17 A dependent manner [32], which suggests a new target for controlling COPD [32].

Airway fibrosis in COPD is usually considered to be the result of long-term airway inflammation. Airway fibrosis, primarily caused by fibroblast differentiation into myofibroblasts, is a direct consequence of the inflammatory response triggered by exposure to inhaled cigarette smoke and leads to the narrowing of small airways [33]. EV-miRNAs alterations could lead to airway fibrosis, which are hallmark processes in COPD. Myofibroblasts have been found to contribute to airway fibrosis by producing extracellular matrix components, including collagenous proteins and α-smooth muscle actin (α-SMA), which give them strong contractile activity [34]. Fujita et al. discovered that exposure to cigarette smoke extract (CSE) can enhance the expression of exosomal miR-210 in bronchial epithelial cells [35]. This, in turn, promotes the conversion of lung fibroblasts into myofibroblasts by targeting autophagy-related 7 (ATG7) [35]. Insufficient expression of ATG7 leads to decreased autophagy, resulting in myofibroblast differentiation in lung fibroblasts [35].Another study also observed that CSE stimulated bronchial epithelial cells to produce miR-21-containing exosomes. The exosomal miR-21 from CSE-treated bronchial epithelial cells could promote myofibroblast differentiation by targeting von Hippel–Lindau protein (pVHL) [36].

### Protease and anti-protease imbalance

Protease and anti-protease imbalance is one of the important pathogenic mechanisms of COPD [37].Proteases can cause damage to the lung parenchyma by breaking down the extracellular matrix, while anti-proteases have the ability to protect it by binding with proteases [38]. Under normal conditions, protease and anti-protease are in equilibrium. When the hydrolytic capacity of protease exceeds the protective capacity of anti-protease, this balance is disrupted and lung parenchyma damage occurs, ultimately leading to the development of COPD [38].The main proteases and anti-proteases involved in the pathogenesis of COPD include neutrophil elastase (NE), matrix metalloproteinase (MMP), andα1-antitrypsin [39]. α-1 antitrypsin, remains the most significant contributor in the pulmonary interstitium. EVs derived from innate immune cells, such as PMNs(polymorphonuclears) and macrophages, exhibit a strong capacity for direct proteolysis. Genschmer et al. found that active neutrophil-derived EVs assisted NE in bypassing the anti-protease protective barrier of the lung and promote extracellular matrix destruction triggering loss of alveolar units leading to emphysema [40].Similarly, EVs are involved in the transportation of α1 antitrypsin. It has been estimated that approximately 1–5% of COPD patients suffer from a deficiency in this protein [41]. Lockett et al. discovered that lung endothelial cells transfer α1-antitrypsin to alveolar epithelial cells through EVs, while cigarette smoke hinders this process by suppressing exosomal activity derived from endothelial cells [42]. Recently, LI et al. identified that exposure of macrophages to tobacco smoke extract (TSE) induced the release of microvesicles with proteolytic activity [43]. Surprisingly, they found that smoke-induced macrophage microvesicles carry significant gelatinolytic and collagenolytic activities, primarily attributed to MMP14 [43], which is involved in the development of emphysema in COPD. The above studies suggest that EVs are involved in regulating the protease-anti-protease system mainly through activation or transport of protease- and anti-protease-related substances.

### Oxidative stress

The imbalance between increased oxidative factors and antioxidant defense mechanisms is referred to as oxidative stress, which plays a crucial role in the development of COPD [44]. Several studies have shown that patients with COPD experience an increased oxidative load [45, 46]. The presence of reactive oxygen species (ROS) in the airways is a major contributor to the development and progression of COPD [47].When the balance between ROS production and antioxidant responses is disrupted, such as by exposure to PM2.5 or cigarette smoke, an accumulation of ROS occurs, leading to oxidative stress [48, 49].The role of EVs in oxidative stress was confirmed by Qiu et al. [50]in a vitro study. A significantly higher level of malondialdehyde, superoxide, and ROS increased in cell with lymphocyte-derived microparticles(LMPs) treatment, while simultaneously inhibiting the production of the antioxidant glutathione [50]. Recent research has shown that there is a close relationship between oxidative stress and mitochondrial damage in COPD, with the two factors interacting [51, 52]. Additionally, exosomes are involved in oxidative stress mainly by affecting mitochondrial function. EVs mediate mitochondrial production of reactive oxygen species in receptor T cells by participating in mitochondrial transfer [53].Adipose mesenchymal stem cell-derived EVs improve macrophage mitochondrial integrity and relieve mitochondrial reactive oxygen stress in macrophages by transporting their mitochondrial components to alveolar macrophages [54]. Thus, EVs ultimately contribute to COPD through mechanism induced by oxidative stress.

### Autoimmune response

COPD is, to some extent, also considered an autoimmune disease [55]. Repeated exposure to cigarette smoke or pathogens activates pattern recognition receptors, such as Toll-like receptors, which in turn activate epithelial cells and innate immune cells like macrophages and neutrophils [55]. This leads to the release of damage-related molecules and subsequently the development of an adaptive immune response in the lungs [55]. Autoantibodies, including anti-elastin antibodies [56], anti-epithelial cell antibodies [57], and tobacco anti-unique antibodies [58], can be detected in the circulation of patients with COPD. Polymeric immunoglobulin receptor-deficient mice develop a progressive COPD-like phenotype spontaneously [59]. Pulmonary macrophages are important immune effector cells that play a critical role in both innate and adaptive immune responses. After brief stimulation of mouse macrophages with ATP, MHC-II-containing EVs are released by the macrophages that mediate antigen presentation and immune activation [60], suggesting a potential role in autoimmune response. Secondly, it has been found that lung macrophages can transport miRNA233 via EVs to various respiratory cells, including lung epithelial cells [19].Additionally, miRNA233 is believed to play a crucial role in regulating the innate immune response in COPD [61]. In addition, phenotypic alterations in lung tissue macrophages are associated with the development and progression of COPD [62]. Wang et al. discovered that EVs originating from airway epithelial cells induced by cigarette smoke altered the phenotype of macrophages and promoted polarization towards M1-type macrophages [63]. The above studies suggest that EVs may participate in the pathogenesis of COPD by transporting relevant immune modulators and regulating relevant immune cells.

### Cell senescence

There is no doubt that COPD is typically found in the elderly and closely associated with aging. In healthy individuals, male forced expiratory volume in the 1st second (FEV1) and forced vital capacity (FVC) reach their peak around the age of 25 and then gradually decline as they get older [64]. Cellular senescence is a state of irreversible growth arrest that can be triggered by either telomere shortening or telomere-independent signals such as DNA damage and oxidative stress [65]. Senescence is characterized by changes in morphology and metabolism, chromatin remodeling, altered gene expression, and the emergence of a pro-inflammatory phenotype known as senescence associated secretory phenotype (SASP) [65].Abnormal cellular senescence in lung tissue is one of the mechanisms involved in the pathogenesis of COPD. Senescent cells, such as alveolar epithelial and endothelial cells, accumulate in the lungs of patients with COPD, resulting in small airway fibrosis and emphysema [66]. Recent studies suggest that senescent cells in the lungs contribute to age-related lung diseases, such as COPD, by releasing SASP factors [67]. EVs released by senescent cells have the ability to transport factors associated with senescence and regulate the phenotype of recipient cells, similar to SASP factors. Thus, EVs secreted by senescent cells are also considered a novel SASP factor. A recent study discovered an increase of EV-miR-21 expression in vitro senescent cells, which potentially triggers the process of cellular aging in nearby cells [68]. Additionally,levels of serum exosomal miR-21 was found to be over-expressed in COPD patients [36].EVs participate in the transport of senescence-associated miRNAs,transmitting cellular senescence which has been further supported by evidence in idiopathic pulmonary fibrosis [69]. Unfortunately, there is currently limited reports regarding the correlation between EVs and cellular senescence in COPD. Further investigation is clearly warranted to conduct additional research.

## Biomarker potential of EVs in COPD

Biomarkers are clinical characteristics that reflect the activity of a disease and fluctuate with its progression, rendering them valuable for diagnosis, monitoring of disease evolution, as well as assessment of therapeutic response [70].EVs are promisingbiomarker candidates due to the high stability of their phospholipid bilayers in bodily fluids and their ability to encapsulate a variety of disease-associated biomolecules. Circulating endothelial microparticles(EMPs) are small membrane vesicles released from endothelial cells in response to stimuli such as inflammatory activation, apoptosis, or injury [71]. They serve as novel biomarkers of endothelial activation and injury. The analysis of circulating EMPs is currently underway to assess endothelial damage in COPD patients and its clinical correlations. A study has found that the level of circulating EMPs increases in smokers with emphysema, which could be useful for identifying early development of the disease [72].Takahashi et al. found that the stable COPD patients had considerably higher levels of EMPs than the non-COPD volunteers [73]. According to another study, CD31(+) EMPs were found to be increased in mild COPD and emphysema; CD62E (+) EMPs were elevated in severe COPD and hyperinflation [74]. The study by Bazzan et al. showed a higher number of alveolar macrophage-derived MVs in the smokers with COPD compared to smokers without COPD and to healthy individuals, which correlated positively with the pack-years of smoking and inversely with lung function expressed as FEV1% (Forced Expiratory Volume in the 1st second percent predicted, reduced FEV1 indicated the degree of airway obstruction) [75].These studies are listed in Table 1.

Some studies have demonstrated that alterations in the circulating miRNA are the physiological responses to COPD development [76]. Thus, circulating miRNAs have the potential to serve as biomarkers for COPD. Emerging evidence suggests that some exosomes contain cell-specific miRNA which have the potential to serve as biomarkers [77–79]. A recent study using exosomal miRNA profiling demonstrated that exosomal miR-122-5p was downregulated among the COPD patients comparing to normal non-smokers and smokers functionally serving as a biomarker [79].Many of the concluded studies are listed in Table 1.

The composition of protein in EVs is also linked to specific cellular functions, indicating that EV proteins have the potential to serve as biomarkers. Koba et al. have utilized next-generation proteomics to identify novel biomarkers in serum EVs [80]. Among them, fibulin-3, a pathogenic matricellular protein in elastic fibers, may serve as a potential biomarker for COPD [80].This discovery implies that circulating EVs protein cargo may serve as biomarkers for COPD. However, there is a dearth of reports that focus on EV protein cargo as biomarkers, and further investigation is required to address this issue.

**Table 1:** Biomarker potential of EVs in COPD

References		Body Fluids	Cohort	Potential Biomarkers
[73]		plasma	stable COPD patients, exacerbated COPD patients, healthy individuals	CD144+ EMPs, CD31+ EMPs, CD62E+ EMPs
[74]		plasma	COPD control subjects	CD31+ EMPs, CD62E+ EMPs
[75]		BALF	smokers with COPD, smokers without COPD, nonsmokers	CD14+ Macrophage microparticles
[77]		plasma	nonsmokers, smoker, COPD patients	upregulated miR-22-3p, miR-99a-5p, miR-151a-5p, miR-320b, miR-320d; downregulated miR-335-5p, miR-628-3p, miR-887-5p, and miR-937-3p
[78]		plasma	COPD patients, healthy individuals	miR-23a, miR-221, and miR-574
[79]		BALF and LungTissue	healthy non-smokers, smokers, patients with COPD or IPF	miR-122-5p
[80]		serum	patients with COPD, healthy controls	fibulin-3

## Therapeutic potential of EVs in COPD

The efficacy of treatments for patients with COPD is currently limited, as they can only alleviate symptoms and prevent exacerbations to a certain extent, but cannot halt the progression of COPD. Mesenchymal stromal cells (MSCs) have been reported to be anti-inflammatory and regenerative. Some studies have suggested that MSC-derived EVs appear to possess the same functions, indicating a novel target for COPD control. Maremanda et al. report that MSCs and MSC-derived EVs protected against cigarette-induced inflammation and mitochondrial dysfunction in a mouse model of COPD [81]. Ridzuan et al. discovered that the intratracheal administration of human umbilical cord mesenchymal stem cell (hUC-MSC)-derived EVs effectively alleviated inflammation in a rat model of cigarette exposure-induced COPD [82].Song’s study revealed that EVs secreted by damaged alveolar epithelial type II (AEC-II) cells can promote the proliferation and migration of MSCs [83]. Additionally, EVs secreted by AEC-II cells increased the expression levels of genes related to mitochondrial synthesis and metastasis [83]. This finding provides a new idea for treating COPD with MSCs. In addition to MSC-derived EVs, other sources of EVs have also been shown to intervene in COPD. Recent study has found that adipose stem cell-derived EVs alleviate cigarette smoke-induced lung inflammation and injury by inhibiting alveolar macrophage pyroptosis [84]. Although there is a lack of studies on the use of these strategies for COPD treatment, some EVs have already been investigatedfor cancer and transplantation treatments in phase I and II trials [85–87]. Some issues still need to be addressed for future clinical application of EVs for COPD treatment.Firstly,there is an inherent issue in acquiring EVs due to the requirement of large amounts of sample and the limitations of obtaining fresh samples in a longitudinal study.Secondly,EVs can be isolated and purified from cell culture supernatant and biological fluids through many methods including ultrafast centrifugation, density gradient centrifugation, immunoaffinity capture,which have their advantages and disadvantages [88–90].But there is no unified standard for EV isolation and characterisation.Additional consideration is the safety behind EV as a novel biomedical products.Some studies suggest that EVs have certain safety [91, 92], but no recognized safety evaluation system has been established.

## Conclusion

In summary, various lung cells secrete EVs that play a crucial pathogenic role in the development of COPD by transporting miRNAs and proteins. The role of EVs in the development of inflammation and protease imbalance in COPD has been partially investigated, but further research is necessary to explore other pathogenic mechanisms. At the same time, EVs are expected to identify more precise biomarkers for COPD, as their phospholipid bilayer packaging allows them to remain stable in various biological fluids of the respiratory system. Although the potential of EVs in treating COPD has been explored in animal models, further research is necessary to determine their clinical applicability. Nevertheless, the outlook for future studies is promising. Some issues related to exosome research require resolution, including the clinical applicability of EV detection, isolation and purification. Further research is urgently needed for clinical application. By investigating the role of EVs in COPD development, a deeper understanding of its pathogenesis can be gained and novel diagnostic and therapeutic methods developed.

## Data Availability

Not applicable to this study.

## Abbreviations

COPD: Chronic obstructive pulmonary disease
EVs: Extracellular Vesicles
WHO: World Health Organization
CPH: China Pulmonary Health
MVBs: Multivesicular bodies
TSG101: Tumor susceptibility gene 101
ISEV: International Society of Extracellular Vesicles
SOCS1: Suppressor of cytokine signaling protein 1
SOCS3: Suppressor of cytokine signaling protein 3
CRP: c-reactive protein
sTNFR1: Soluble tumor necrosis factor type I receptor
IL-6: Interleukin-6
CSE: Cigarette smoke extract
CYR61: Cysteine rich 61
TNFα: Tumor necrosis factor-alpha
MCP-1: Monocyte chemotactic protein-1
ICAM-1 or CD54: Intercellular adhesion molecule-1
α-SMA: α-smooth muscle actin
ATG7: Autophagy-related 7
Pvhl: Von Hippel–Lindau protein
NE: Neutrophil elastase
MMP: Metalloproteinase
PMNs: Polymorphonuclears
TSE: Tobacco smoke extract
ROS: Reactive oxygen species
LMPs: Lymphocyte-derived microparticles
SASP: Senescence associated secretory phenotype
EMPs: Endothelial microparticles
BALF: Broncho-alveolar lavage fluid
FVC: Forced vital capacity
FEV1: Forced expiratory volume in the 1st second
FEV1%: Forced expiratory volume in the 1st second percent predicted
MSCs: Mesenchymal stromal cells
hUC-MSC: Human umbilical cord mesenchymal stem cell
AEC-II: Alveolar epithelial type II
